# Supplementary figures and images for: The E2 glycoprotein holds key residues for Mayaro virus adaptation to the urban Aedes aegypti mosquito
Source: PLoS Pathog. 2023 Apr 5;19(4):e1010491. doi: 10.1371/journal.ppat.1010491 (PMC10109513; doi:10.1371/journal.ppat.1010491)

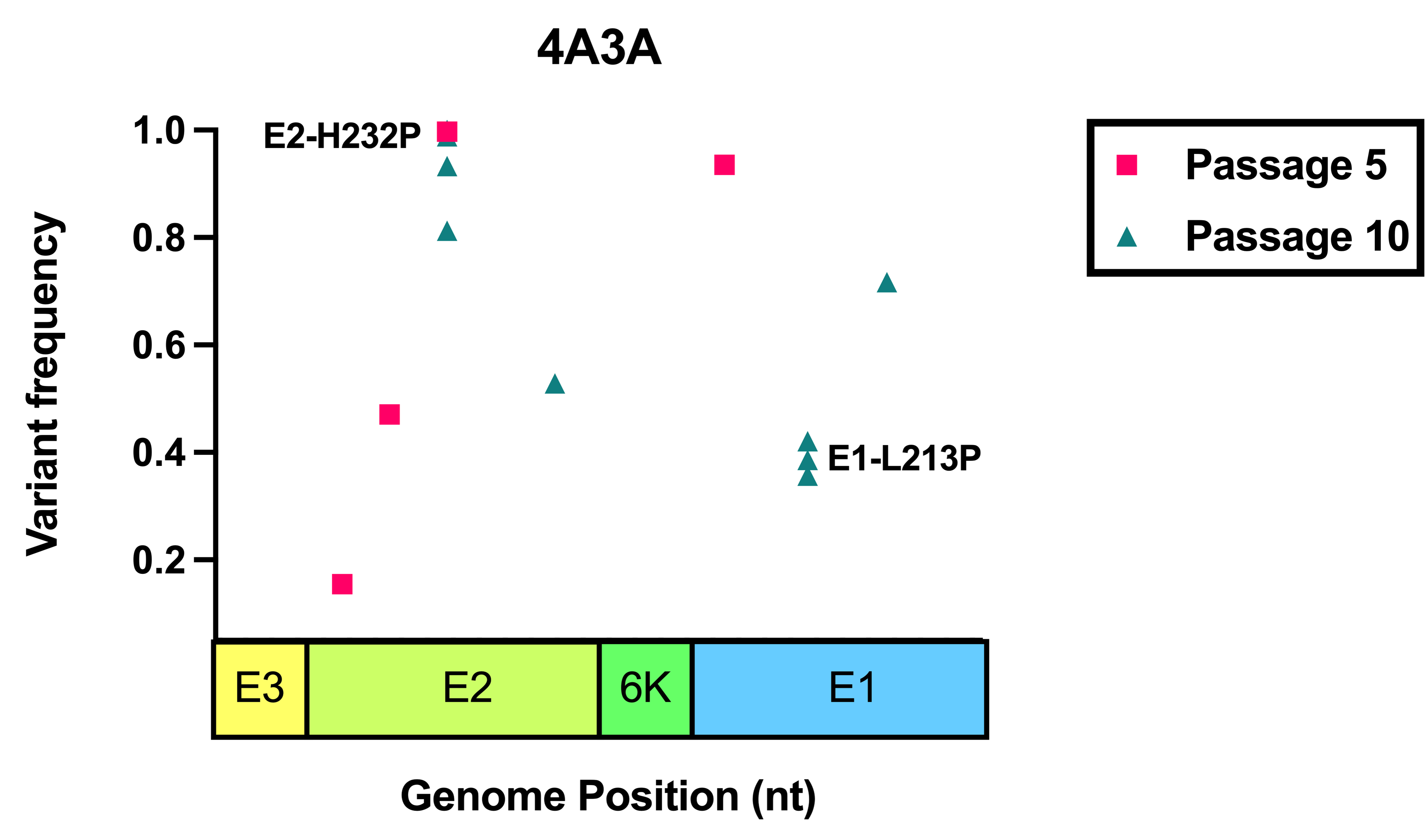

Supplement: S1 Fig — Experimental evolution was performed using traditional serial passaging in 4a-3A cells. We serially passaged MAYV at a MOI of 0.01 for a total of ten passages. Following one, five, and ten passages, the viral RNA was sequenced using Illumina NGS to identify potentially adaptive mutations. No high frequency variants were identified following one passage, and as such are not depicted here. (TIF) [file ppat.1010491.s001.tif]

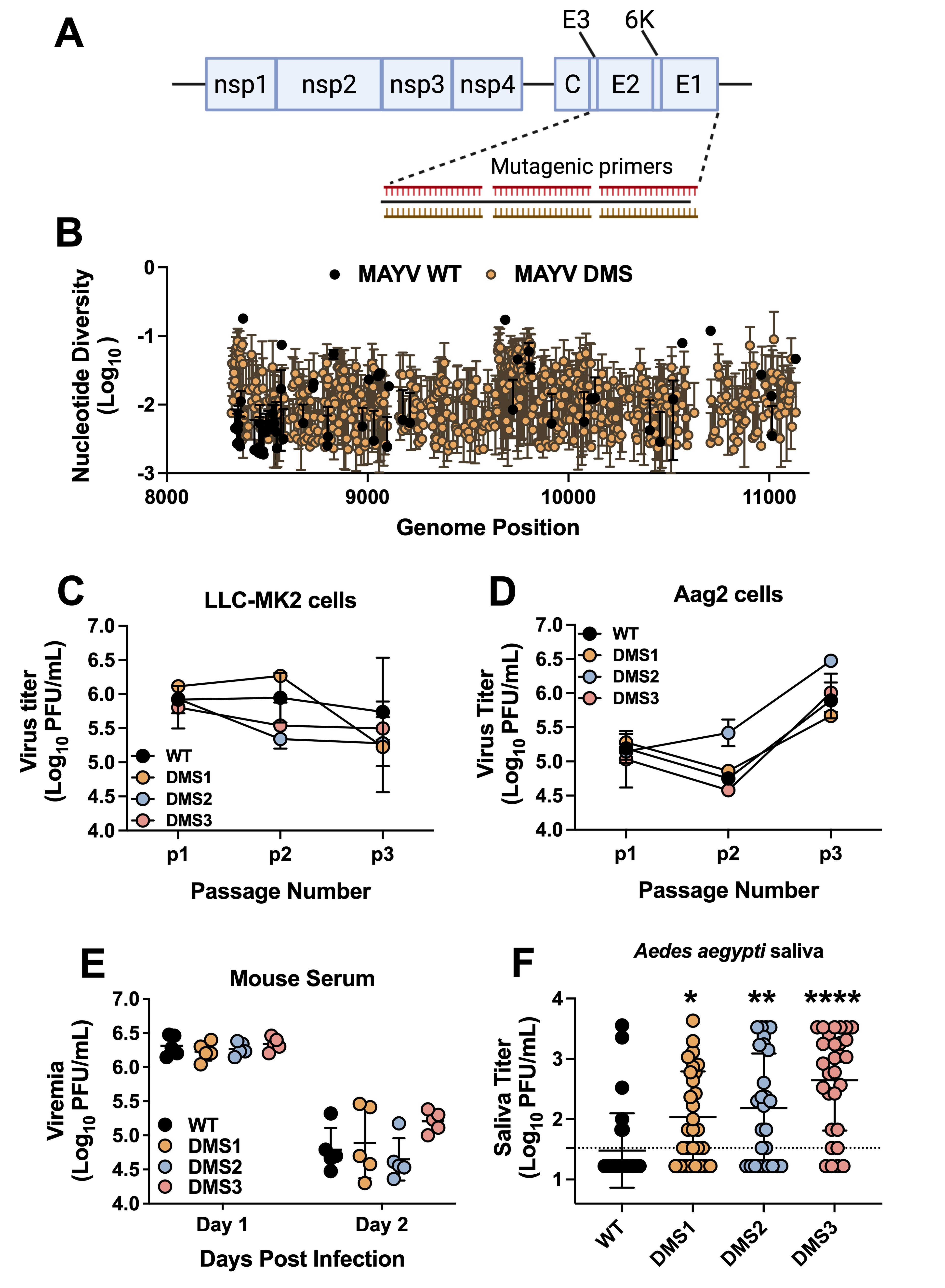

Supplement: S2 Fig — A. Genome organization of MAYV DMS viruses. Created with BioRender.com. B. Non-synonymous nucleotide diversity for wild-type (WT) and MAYV DMS. The three independent MAYV DMS populations were combined to aid visualization. C-D. Three passages were performed in vitro at an MOI of 0.01 in LLC-MK2 (monkey kidney; C) and Aag2 (Aedes aegypti mosquito; D). E. Viremia of WT MAYV and DMS populations in mice during passaging. F. Viral titers in saliva of WT MAYV and DMS populations after passage in Ae. aegypti mosquitoes. Statistical analysis: * = p<0.05; ** = p<0.01; **** = p<0.0001 (one-way ANOVA with Dunnett’s correction). (TIF) [file ppat.1010491.s002.tif]

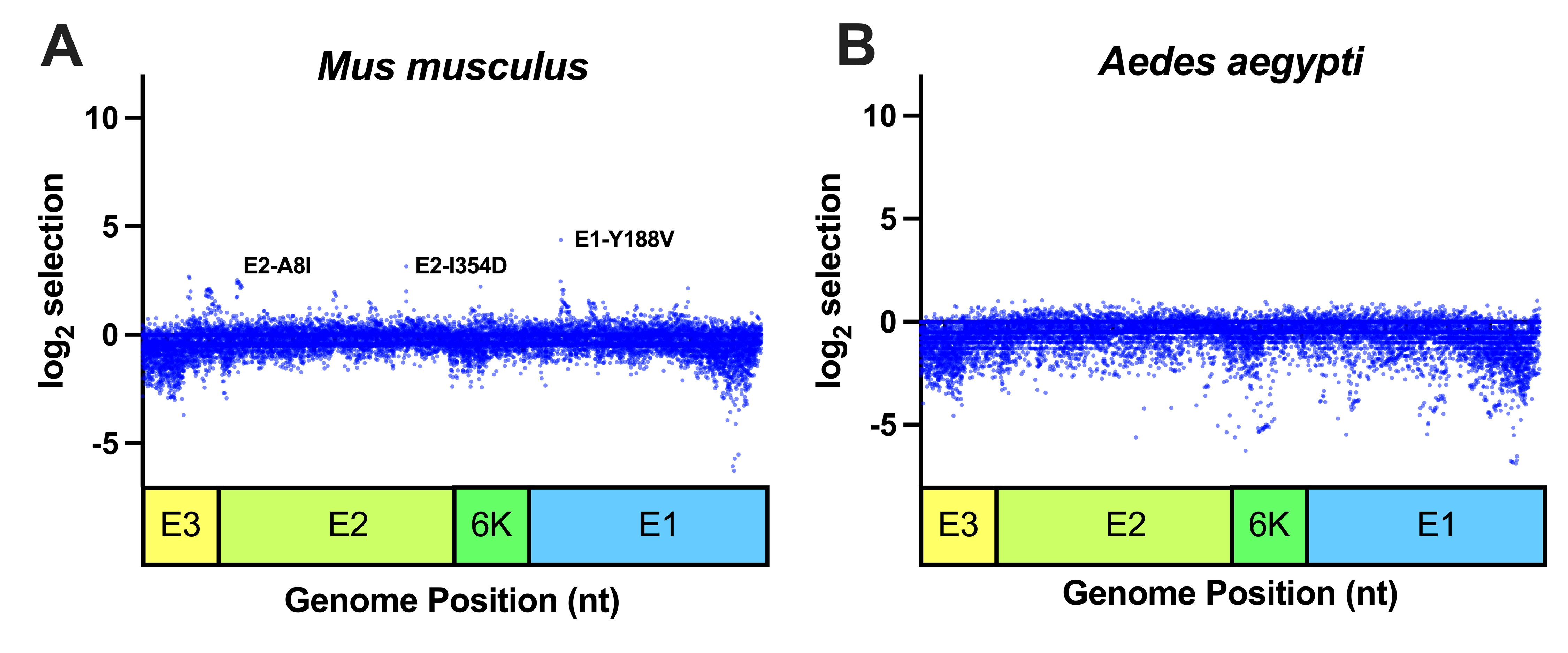

Supplement: S3 Fig — A-B. The three MAYV DMS populations, along with WT MAYV, were used to perform three passages in mice (A) and Ae. aegypti mosquitoes (B). Following passage, the viral RNA was sequenced, and selection analyses were performed to identify enriched variants. The top three variants based on selection strength, are presented for mice (A). Given the low level of enrichment observed in Ae. aegypti mosquitoes, we do not present individual variants. (TIF) [file ppat.1010491.s003.tif]

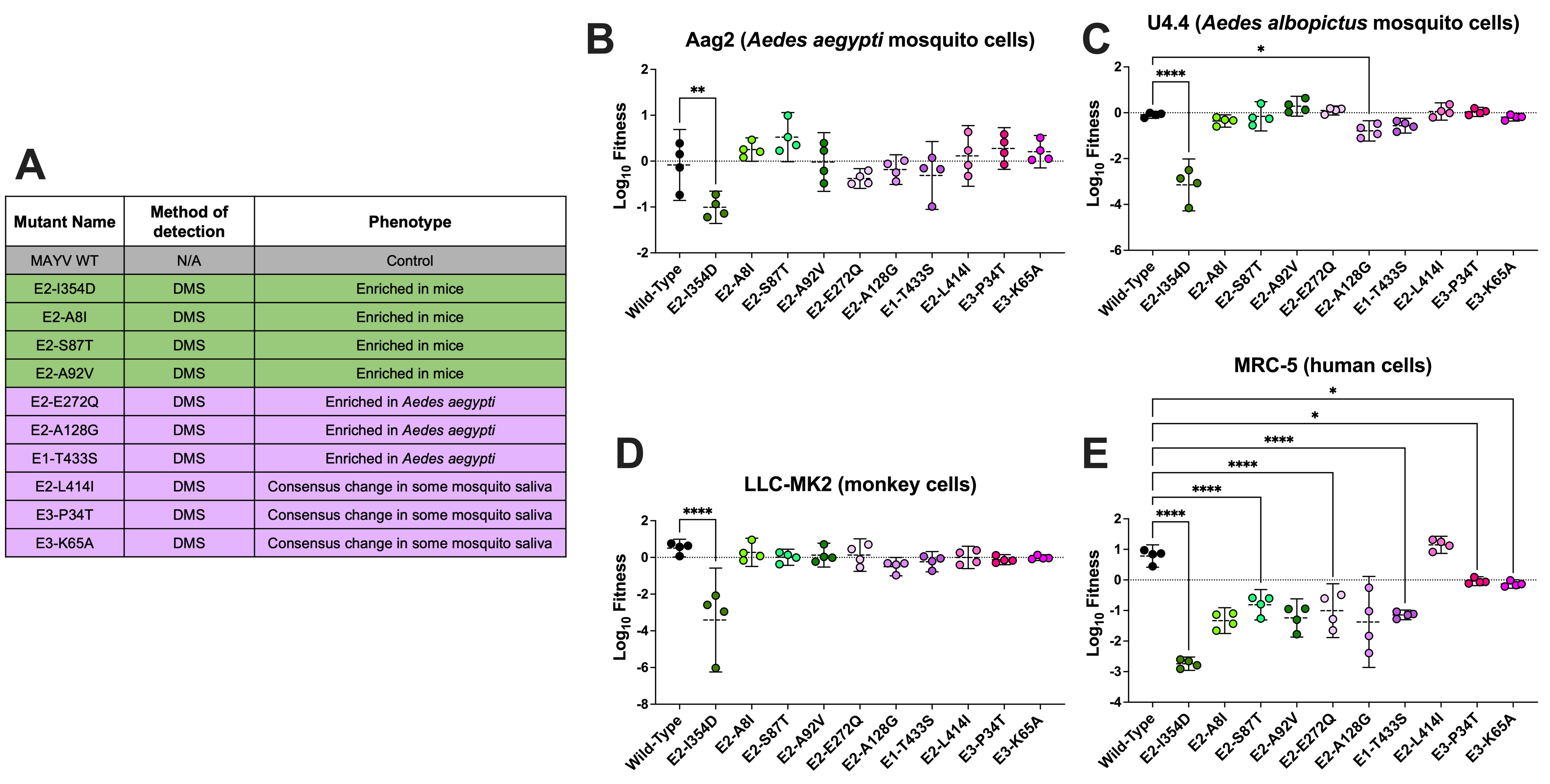

Supplement: S4 Fig — A. List of the viruses used for the competition assays. All mutations were identified from deep mutational scanning data, and the phenotypes observed for the different viruses are indicated. The wild-type data—included as a comparison—is the same as is presented in Fig 2 in the main text. B-E. Competition assays in Aag2 (B), U4.4 (C), LLC-MK2 (D), and MRC-5 (E) cells. Cells were infected at a MOI of 0.01 using a 1:1 ratio based on PFUs for each mutant or WT with a genetically marked MAYV competitor virus. Viral supernatants were harvested at 72h post-infection for Aag2 and 48h post-infection for the other cell lines. Replication of WT and mutant viruses was assessed by RT-qPCR using specific probes labeled with different fluorophores. Log10 fitness was calculated by normalizing replication of each virus against a genetically marked reference virus. The mean of 4 independent experiments is represented with standard deviation. Statistical analysis: * = p<0.05; ** = p<0.01; **** = p<0.0001 (one-way ANOVA with Dunnett’s correction). Consensus change in some mosquito saliva means that the mutation was found at above 50% in one mosquito saliva sample: E2 L414I was found at 89%, E3-P34T was found at 99.7%, and E3-K65A was found at 99.7%. (TIF) [file ppat.1010491.s004.tif]

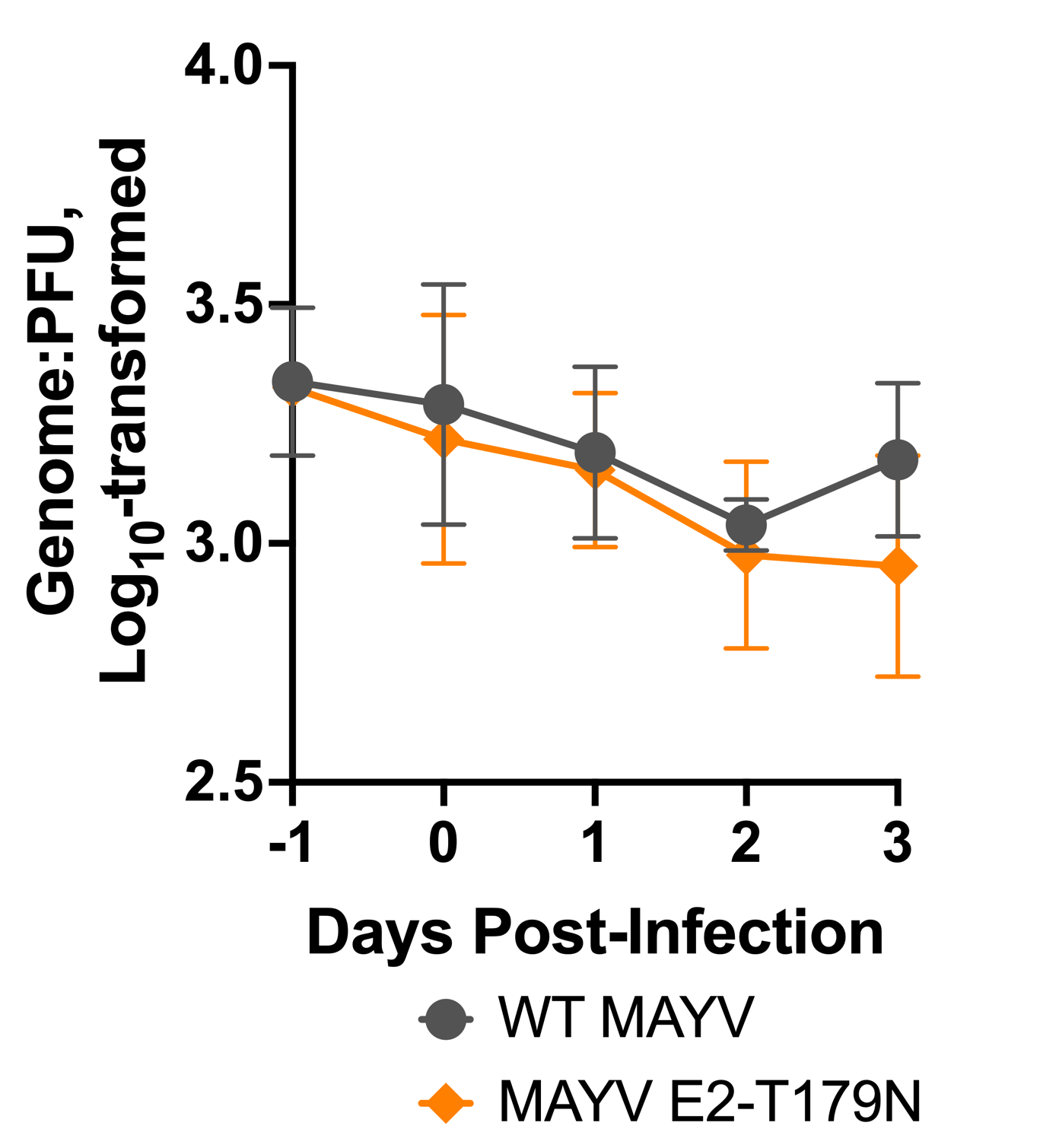

Supplement: S5 Fig — Aag2 cells were infected with either WT MAYV or MAYV E2-T179N at a MOI of 0.1, and genome:PFU ratios were measured each day post infection via RT-qPCR and plaque assay. The -1 day post infection represents the genome:PFU ratio of the inoculum used for the infection. Genome:PFU ratios represented are log10-transformed. The means of two independent experiments are represented, and error bars represent standard deviation. Statistical analysis: non-significant (two-way ANOVA with Šídák’s multiple comparisons test). (TIF) [file ppat.1010491.s005.tif]

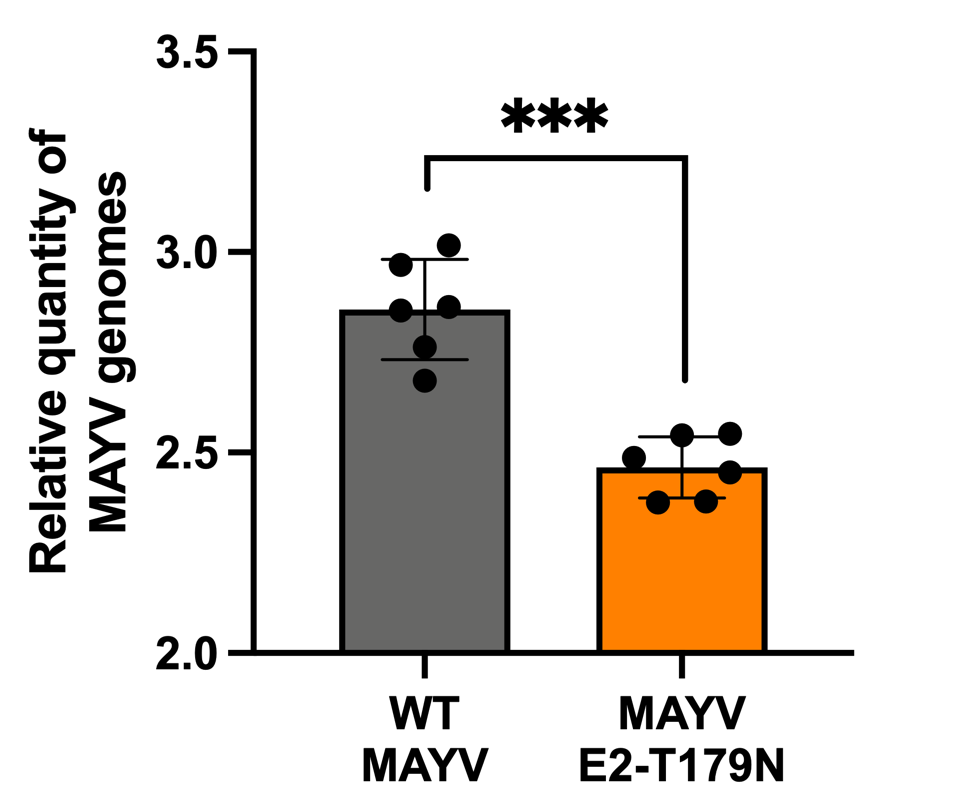

Supplement: S6 Fig — Binding assays of WT MAYV and MAYV E2-T179N were performed by inoculating chilled Aag2 cells with virus at a MOI of 0.1 prior to adsorption at 4°C. Unbound virus was washed away, and bound virus was quantified by RT-qPCR using RNA extracted from the inoculated cells. The relative quantities of MAYV genomes were determined by normalizing to the Ct values of a housekeeping gene and the Ct value of virus in the inoculum. Data represent the means of two independent experiments. Y-axis is log-transformed for clearer visualization. Statistical analysis: *** = p<0.002 (unpaired t-test). (TIF) [file ppat.1010491.s006.tif]

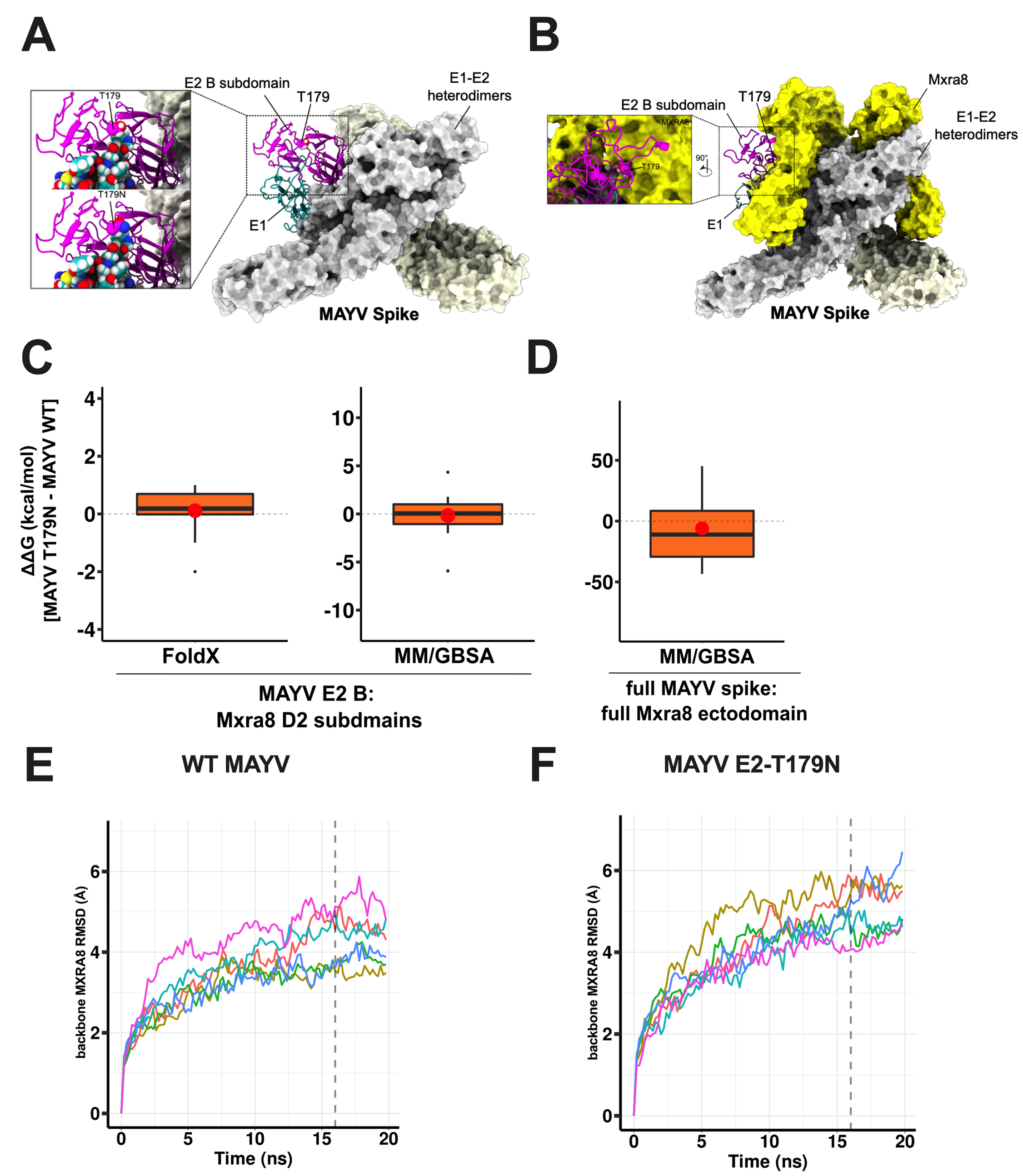

Supplement: S7 Fig — A. MAYV spike structure (PDB ID: 7KO8) composed of three E1-E2 heterodimers. Only the E1-E2 ectodomains are presented. For clarity, we showed only one heterodimer (E1 in green and E2 in magenta) in cartoon representation, and the other two are represented as a surface. Zoomed-in views of T179 and the T179N mutation are shown in insets. T179, T179N and the E1 protein are represented in spheres. B. MAYV spike in complex with three copies of the human Mxra8 receptor (in yellow) obtained from the last frame of the 20 ns molecular dynamics simulations. For clarity, only one E1-E2 heterodimer is represented as a cartoon. Inset shows a side view of the interface between the E2 B subdomain and the Mxra8 receptor. T179 is shown in spheres. All images were generated using the ChimeraX software. C. Binding free energy changes (ΔΔG in kcal/mol) of 12 MAYV E2 B:Mxra8 D2 subdomain complexes (n = 12) upon MAYV T179N mutation using FoldX and MM/GBSA methods. ΔΔG was obtained by subtracting the ΔG estimated for T179N MAYV:Mxra8 interaction from the ΔG obtained for WT MAYV:Mxra8 interaction. MMGBSA calculations were performed from 2 ns molecular dynamics simulation (in triplicate) of each complex. In none of the applied methods did the estimated ΔΔG significantly differ from zero (p > 0.05—one sample t-test). The box plot central line indicates the median and the red circle indicates the mean. D. Binding free energy changes (ΔΔG in kcal/mol) of the full trimeric MAYV spike in complex with the full Mxra8 ectodomain upon MAYV T179N mutations using MM/GBSA method. The calculations were performed from a quintuplicate of 20 ns molecular dynamics simulations (n = 5) (see methods for details). ΔΔG was obtained by subtracting the relative ΔG estimated for T179N MAYV:Mxra8 interaction from the ΔG obtained for WT MAYV:Mxra8 interaction. The estimated ΔG did not significantly differ from zero (p > 0.05—one sample t-test). The box plot central line indicates the median, and the red circle indica [file ppat.1010491.s007.tif]
