# Supplementary material for: The E2 glycoprotein holds key residues for Mayaro virus adaptation to the urban Aedes aegypti mosquito
Source: PLoS Pathog. 2023 Apr 5;19(4):e1010491. doi: 10.1371/journal.ppat.1010491 (PMC10109513; doi:10.1371/journal.ppat.1010491)
Supplement: Attachment — Submitted filename: Exp07_response_letter_The_E2_glycoprotein_holds.docx [file ppat.1010491.s012.docx]

Dear Dr. Heise,

Thank you for your time and thoughtful comments on our manuscript “The E2 glycoprotein holds key residues for Mayaro virus adaptation to the urban Aedes aegypti mosquito" (PPATHOGENS-D-22-00591) to *PLOS Pathogens*. We also appreciate the extra time you allotted us to provide thoughtful responses to the reviewers. We are grateful to the reviewers and you for providing comments and suggestions that improve the rigor and scientific merit of our experiments. Please see below how we have addressed each comment by either performing additional experiments or modifying language in the manuscript to better communicate our results. The editor and reviewer comments are in quotations, and our responses follow each comment in bold.

**Editor comments:**

“The manuscript is being returned with three reviews. While each of the reviewer's comments should be addressed, particular attention should be focused on the following issues:

**We thank the editor and reviewers for helping us focus our attention on these three critical points.**

“(1) Both R1 and R2 raised concerns about the virus binding and replication data, including the role of Mxra8, other entry receptors, and the rigor of the experiments (e.g., the need for appropriate positive and negative controls). The authors should provide additional data to explain differences in binding and replication between WT and E2 T179N viruses.”

**We now present data explaining the impact of the mutation on binding and replication in fibroblasts—WT MAYV appears to be more dependent on Mxra8 than the mutant virus. We modified language in the manuscript to make it clear that WT MAYV serves as an internal positive control since it is known to enter through Mxra8 (PMID: 29769725) and should therefore demonstrate reduced replication in 3T3 Mxra8 KO cells, which we observed (Fig. 4D). We now show that Mxra8 and ApoER2 may contribute to the attenuation of the mutant.**

**Towards understanding the mechanisms enhancing replication in mosquito cells, we also performed a one-step growth curve in Aag2 cells to investigate virus release after one cycle of replication (Figure 3F). We observed a greater titer of the mutant after a single round of replication compared to WT MAYV. These studies help shed light on the mechanism by which the virus has increased fitness in *Ae. aegypti* cells.**

“(2) Address the concerns of R3 regarding statements of MAYV emergence and the emergence potential of MAYV E2 T179N, as well as other speculative components of the manuscript.”

**We have addressed R3’s concerns by modifying statements particularly about MAYV emergence at the end of the introduction, and speculative comments without any supporting data have been removed, especially throughout the results section**

(3) Issues with reproducibility in mosquito transmission data should be addressed.

**The reproducibility in the mosquito transmission data has been addressed by performing two additional independent transmission experiments in a different colony of *Aedes aegypti* mosquitoes. The studies were performed at Virginia Tech by different experimenters instead of The Pasteur Institute, which provides a further level of rigor and reproducibility. The trends are consistent between the two studies: significant results between the original studies and additional studies for transmission rates of infected or disseminated mosquitoes (lines 377-432). We have also included the sample sizes for each independent experiment in Figure 5 and included a greater overall sample size by performing the two new transmission studies.**

**Part II – Major Issues: Key Experiments Required for Acceptance**

Reviewer #1: “…the authors must show the fitness of MAYV T179N mutant virus derived from serial passaging in U4.4 cells. In comparison to replication in Aag2 cells, include data on the binding and replication of MAYV WT and MAYV T179N mutant virus in U4.4 cells. Fitness studies indicate that mutation may facilitate the transmission of MAYV by A. albopictus mosquitoes.”

**We have now included a growth curve of the wild type and mutant viruses in U4.4 cells (Figure 3B). We did not perform binding assays because the focus of our vector competence studies was adaptation to *Aedes aegypti;* however, in future studies, we intend to explore binding to U4.4 cells and transmission studies in *Aedes albopictus*.**

“2. In insect cells, Mxra8 is not a receptor for alphaviruses. In the absence of any structural differences it is intriguing why there was decreased binding of MAYV T179N to MRC-5 cells. The authors will need to explore importance of other receptors such as NRAMP2, VLDLR (Rose et al., Cell Host Microbe 2011, 10(2)97-104; Clark et al., Nature 2022, 602; 475–480) in binding of MAYV to MRC-5 and Aag2 cells. NRAMP2 is required for Sindbis virus binding, entry, and infection in both mammalian and insect cells. Caluwe et al., Front Microbiol 2021, 12: Art 615165 have reported that CD147 complex proteins are involved in the entry of CHIKV and MAYV.”

**Thank you for this comment which helped us guide how we explore the attenuating mechanism of MAYV E2-T179N in a mammalian environment. We performed an alternative analysis of the growth curve experiments in WT 3T3 and 3T3 Mxra8 KO cell lines and conclude that MAYV E2-T179N is less dependent on Mxra8 than is WT MAYV. We also screened a panel of putative MAYV receptors including SLC1A5, hTIM4, hVLDLR, and hApoER2 isoforms 1 and 2 and found that WT MAYV has significantly increased replication in the presence of hApoER2 and Mxra8 while MAYV E2-T179N does not. These results show that the reduced dependence of MAYV E2-T179N replication on hMxra8 and hApoER2 are associated with replication attenuation in mammalian cells (lines 303-346).**

“3. Structural studies did not reveal any differences between E2 T179N and WT E2 protein binding to Mxra8 (Fig 4C,D and E). However, replication of MAYV T179N was reduced in Mxra8 3T3 KO cells compared to 3T3 cells infected with WT virus, which indicate the importance of Mxra8 in MAYV replication. Data showing the binding of MAYV T179N to WT and Mxra8 KO 3T3 cells must be included.”

**Thank you for addressing this important mechanistic result. We have presented a different approach to analyzing the replication data of MAYV WT and MAYV E2-T179N in both WT 3T3 and Mxra8 KO lines. Please view Figure 4F, where we present the reduction in titer for WT MAYV and MAYV E2-T179N from WT 3T3 cells to 3T3 Mxra8 KO cells. We observed a smaller reduction in titer for MAYV E2-T179N compared to WT MAYV when the viruses replicated in 3T3 Mxra8 KO lines as compared to WT 3T3 cells. Contrary to our previous conclusions, this analysis indicates Mxra8 is at least in part responsible for the attenuation of MAYV E2-T179N in mammalian systems (lines 319-324). We explained more clearly that MAYV E2-T179N still uses Mxra8 during infection, only less effectively than WT MAYV (lines 318-322). However, when we performed binding assays on WT 3T3 and 3T3 Mxra8 KO cells, we observed no differences in the reduction of binding between viruses (Fig. 4C). We intend to look at potential differences in internalization and endosomal fusion via Mxra8 in future studies.**

Reviewer #2: “1. While most of the conclusions are supported by the data, there are several exceptions where this is not the case. Notably:
a. The statement: “However, using this model, the absence of Mxra8 induced a similar reduction of viral replication of both WT and E2-T179N MAYV, confirming our in silico analyses showing that the reduced viral binding of E2-T179N MAYV is Mxra8-independent” is not supported by the data shown in Fig 4F. MAYV E2-T179N 3T3 Mrxra8 KO titers appear lower at most/all time points compared to MAYV WT 3T3 Mrxra8 KO. The legend states that statistical tests showed no differences, but this is hard to believe given that the error bars for the MAYV E2-T179N 3T3 Mrxra8 KO titers do not overlap with any of the other groups on days 1, 2, 3 and 4. Wouldn’t the data shown suggest that binding of MAYV E2 T179N is in fact at least partially Mxra8 dependent? Also missing are positive and negative controls for this experiment.”

**We thank the reviewer for drawing our attention to a key difference we missed previously, which helped us identify a lesser dependence on Mxra8 for the mutant. We have now compared the differences between WT and MAYV E2-T179N for WT 3T3 and 3T3 Mxra8 KO cells and found a greater reduction in titer for the WT virus compared to the mutant virus in the absence of Mxra8. We present these data in Fig. 4 and in the results section from lines 313-324.**

“b. Scientific rigor is lacking for Fig 5 which does not show Ns for each of the three experiments, which could help the reader evaluate whether the higher transmission rate of 60% in one MAYV E2-T179N replicate is an artifact of smaller cohort size and therefore more prone to stochastic effects, especially since transmission rates in the other 2 replicates appear much lower.”

**We have now added Ns for each experiment. Furthermore, we have now repeated the vector competence studies with a different mosquito population and at a different institute. Overall, the results are very similar between the two studies: WT MAYV infects mosquitoes better, but of the mosquitoes that developed a disseminated infection—and to a lesser extent any infection—MAYV E2-T179N is able to enter the saliva more effectively. We have added the Ns for all three biological replicates to Figure 5 and also to the two additional biological replicates we performed with higher total Ns for each replicate. We also note this in the figure legend for Figure 5.**

“c. Since mice inoculated with MAYV E2-T179N had a nearly identical pattern of footpad swelling to WT that was delayed by 1 day, the conclusion “These results are consistent with E2-T179N causing an attenuation of MAYV in the mammalian host” is not entirely supported. Delayed, perhaps, but clinical disease in the MAYV E2-T179N mice as assessed by swelling does not seems quantitatively different from WT, which doesn’t support use of ‘attenuation’.”

**We agree with the reviewer. We have updated the text at lines 454-456 to “These results demonstrate that MAYV E2-T179N replicates to lower levels and causes less severe tissue pathology compared to WT MAYV in the mammalian host.”**

“d. For full transparency, the scores of mice selected as ‘representative’ for images 1 and 2 in Fig 6E should be shown.”

**We appreciate this useful suggestion, and have added the exact scores for the images we present to the figure legend for Figure 6.**

“2. Use of the words ‘mosquito’ and ‘mouse’ in several places, while data are being described from mosquito cells and mouse cells, is misrepresentative of the systems in which the experimental evolution/DMS studies were performed. For example:

**We have updated each figure legend as suggested.**

a. Fig 2: Clarify that Aag2 and U4.4 are mosquito cell lines and not mosquitoes (as text in parentheses seems to indicate).”

**Thank you for catching this misleading detail. We have clarified that these are mosquito cells lines in Figure 2.**

“b. Fig S4: Figure heading; clarify that it was mosquito and mouse cells, not entire organisms.” And “c. P13: ‘We also evaluated the fitness of mutations enriched following passage through mice and mosquitoes and observed’…should be mouse and mosquito cells.”

**We are sorry for the lack of clarity. These were actually done in the living organism, not cell lines. We have clarified in the figure, the figure legend, and the text at lines 169-170.**

“3. The Mxra8 data seems a bit of a distraction from the central message of the manuscript in that: 1) it does not explain the less efficient binding of MAYV E2-T179N mutant to human cells, 2) the same phenotype was observed in mosquito cells, and 3) it is unknown known (although probably likely) whether MAYV uses Mxra8 as a primary entry receptor.”

**Based on the reviewer’s previous suggestion, we now have evidence that Mxra8 likely contributes to the mutant’s attenuation (Fig. 4). We plan to dissect these mechanisms in future studies.**

Reviewer #3: “A few other key points of concern (for items throughout the manuscript) are noted below for the authors to consider.

1) In the introduction, the authors tend to overstate the risk of MAYV emergence. As these are all speculative statements, it is suggested that the authors temper these statements with modifiers such as “may” lead to emergence, or “limited MAYV circulation”, etc. In several places, it already sounds like a huge problem; there is no need to try to oversell this preliminary work.”

**We agree with the reviewer, and have modified or removed statements that oversell the mutation’s effect on emergence, particularly at the end of the introduction.**

“2) While some level of speculation is ok in the discussion, the authors really should consider removing most of their “speculations” throughout the results section. For each section, they report their results and then say “this is likely due to…” or “this likely leads to…” (or similar sentiments). Since no work was performed to elucidate mechanisms, it is not really appropriate to add such wild speculation without any supporting data. Provide the data you obtained, state more work is needed to understand the mechanisms, and leave it at that. This has something of the feel of a dissertation rather than a scientific publication.”

**Thank you for offering up this comment as it helps us focus our conclusions on what our data support. We have removed speculative statements about mechanisms throughout the results section, and/or have acknowledged the need for further experiments.**

“3) While perhaps slightly eluded to in the discussion, the authors really fail to mention the role of the remaining viral “backbone” in contributing to the effects of any specific point mutations. Thus, STRAIN is incredibly important and the authors need to mention this. As an example, they refer to CHIKV mutation that leads to altered vector species competence yet they failed to state that this same mutation had no effect in the context of a CHIKV backbone from a different genotype. Asian genotype strains were not affected in the same way as ECSA genotype strains by the same mutations so introduction of this single mutation into any particular clone may not result in a generalizable outcome. This needs to be addressed in the discussion as well particularly since the DMS work focused only on the glycoprotein sections of the genome.”

**We thank the reviewer for this comment. We have mentioned our use of a single MAYV strain as a limitation in the discussion (lines 570-572), as suggested. We have also pointed out that a CHIKV mutant with the same mutation produced slightly conflicting results, which further supports the impact of strain and virus-specific impacts of any given mutation.**

“4) There are a few places in the results where the authors seem to discount “contrary” results and focus only on those data that support their speculations. One example is discounting the finding that “peak footpad swelling was similar between the groups”. This seems to later be ignored in the speculative part of this section. Similarly, it is concerning that there is so little reproducibility in mosquito transmission data (15-60% is huge variation!) yet this concern is not addressed. If the 15% set is considered, this is not significantly different from the control findings. The authors need to use caution in failing to include all the results in their interpretations. Let the data speak for itself and include it all in your discussion.”

**We have updated the text in various places to let the data speak for themselves, especially outside of the discussion. We have also repeated the mosquito experiments with a different mosquito population and at a different institution to increase the rigor of this finding.**

**Part III – Minor Issues: Editorial and Data Presentation Modifications**
Please use this section for editorial suggestions as well as relatively minor modifications of existing data that would enhance clarity.

“Reviewer #1: 1. Fig S4 – please clarify the meaning of some mouse serum and mouse saliva?”

**We have updated the table (Fig. S4A) and the figure legend to clarify. The mouse data we all enriched so we made that consistent. The mutants identified in mosquitoes were selected on a different basis, so we further clarified this selection and their frequency in mosquito saliva in the figure legend.**

2. The E2 mutation T179N resulted in attenuation in MAYV infections in mice. It will be interesting to examine whether the mutation also results in attenuation of CHIKV infections in mice.

**We agree this would be interesting to study, and plan to explore the impact of this mutation on CHIKV in mice and mosquitoes in future studies.**

Reviewer #2: 4. The introduction and results sections lack details on how the DMS works and how it was performed. This makes it difficult for a reader unfamiliar with references 36-38 to understand this approach. Even a brief background/rationale would be helpful.

**Thank you for pointing this out. We have now provided a brief background and rationale for using DMS to orient readers. Line 154: “DMS is a powerful approach to create a diverse viral population that theoretically contains all possible amino acid substitutions at each site within a given genomic region. Following selection in an environment, deep sequencing is performed, and computational analyses then identify sites that are enriched or decreased.”**

5. The rationale for use of Anopheles cells would enable the reader to understand whether the idea was to determine whether MAYV could adapt to multiple mosquito genera (i.e. ref. 35?), or whether this was intended as a negative control, especially since the data from those cells are sort of sidelined by relegation to Figure S1 but nowhere else.

**We have now added additional information on our rationale for including these cells (lines 136-138): “Our rationale for using Anopheles cells was based on a recent report showing them to be competent for MAYV.” We focused on *Ae. aegypti* here because it is the vector for most important arboviruses but would like to look at these potential alternative vectors in future studies.**

6. Fig S3B: It is unclear which dots show the amino acids indicated.

**We have now removed the individual variants from S3B since it is not possible to specify specific dots. We have also noted this in the figure legend: “Given the low level of enrichment observed in Aedes aegypti mosquitoes, we do not present individual variants.”**

7. P8 ‘suggesting a mammalian-specific attenuation’; a bit of a stretch given just 2 cell types were evaluated, suggest tempering language.

**We have removed this statement.**

8. Replacing the labels ‘1’ with ‘WT’ and ‘2’ with ‘E2-T179N’ would make Fig6E more readable.

**You are absolutely right—we have changed the panel labels to detail the groups represented in Figure 6E.**
