# Supplementary material for: The E2 glycoprotein holds key residues for Mayaro virus adaptation to the urban Aedes aegypti mosquito
Source: PLoS Pathog. 2023 Apr 5;19(4):e1010491. doi: 10.1371/journal.ppat.1010491 (PMC10109513; doi:10.1371/journal.ppat.1010491)
Supplement: Attachment — Submitted filename: response_letter_The_E2_glycoprotein_holds_resub.docx [file ppat.1010491.s013.docx]

Dear Dr. Heise,

Thank you for your time and thoughtful comments on our manuscript “The E2 glycoprotein holds key residues for Mayaro virus adaptation to the urban Aedes aegypti mosquito" (PPATHOGENS-D-22-00591) to *PLOS Pathogens*. We are grateful to the reviewers and you for providing comments and suggestions that improved the manuscript.

**Editor comments:**

(1) Please confirm IBC approval for the experiments reported in this manuscript.

(2) Please provide more detailed information about the biosafety practices used during the course of these experiments.

**We have added additional information in the methods under the heading “Biosafety Protocols” to address both comments.**
